# Comparison of zotarolimus-eluting and sirolimus-eluting coronary stents: a study from the Western Denmark Heart Registry

**DOI:** 10.1186/1471-2261-12-84

**Published:** 2012-10-02

**Authors:** Michael Maeng, Lisette Okkels Jensen, Anne Kaltoft, Hans-Henrik Tilsted, Evald Høj Christiansen, Per Thayssen, Morten Madsen, Henrik Toft Sørensen, Jens Flensted Lassen, Leif Thuesen

**Affiliations:** 1 Department of Cardiology, Aarhus University Hospital, Skejby, Brendstrupgaardsvej 100, 8200, Aarhus N, Denmark; 2 Department of Cardiology, Odense University Hospital, Odense, Denmark; 3 Department of Cardiology, Aarhus University Hospital, Aalborg Hospital, Aalborg, Denmark; 4 Department of Clinical Epidemiology, Aarhus University Hospital, Aarhus, Denmark; 5 Department of Epidemiology, Boston University, Boston, MA, USA

**Keywords:** Zotarolimus, Sirolimus, Stent, Mortality, Stent thrombosis, Diabetes mellitus

## Abstract

**Background:**

We evaluated the effectiveness and safety of a zotarolimus-eluting (ZES) versus a sirolimus-eluting (SES) coronary stent in a large cohort of patients treated with one of these stents in Western Denmark.

**Methods:**

A total of 6,122 patients treated with ZES (n=2,282) or SES (n=3,840) were followed for up to 27 months. We ascertained clinical outcomes based on national medical databases.

**Results:**

Incidence of target lesion revascularization (no. per 100 person-years) was 5.3 in the ZES group compared to 1.9 in the SES group (adjusted hazard ratio (HR)=2.19, 95% confidence intervals (CI): 1.39-3.47; *p*=0.001). All-cause mortality was also higher in the ZES group (ZES: 6.3; SES: 3.3; adjusted HR=1.34, 95% CI: 1.05-1.72; *p*=0.02), while stent thrombosis (ZES: 1.2; SES: 0.5; adjusted HR=1.98, 95% CI: 0.75-5.23; *p*=0.14) did not differ significantly.

**Conclusions:**

In agreement with previously published randomised data, this observational study indicated that the ZES was associated with an increased risk of death and TLR in a large cohort of consecutive patients.

## Background

Introduction of first-generation drug-eluting stents (DESs) more than halved the need for target lesion revascularization (TLR) after coronary stent implantation [1-6]. However, safety concerns have been raised about the first-generation DESs, especially regarding an increased risk of late stent thrombosis (>30 days after stent implantation) [4,7]. The effective inhibition of in-stent neointima formation by the first-generation DESs may reduce neointimal coverage of stents, leaving stent struts as a nidus for late stent thrombosis. Late stent thrombosis has also been associated with late-acquired malapposition of stent struts [8] and late vascular inflammation (beyond that associated with the initial vascular injury and subsequent healing) [9], both of which may be related to either the type of drug eluted or the polymer coating of the stents.

Since the second-generation fast-release zotarolimus-eluting Endeavor™ stent (ZES) has been shown to induce relatively uniform and complete neointimal coverage of the stent struts and to have a lower incidence of late-acquired incomplete stent apposition [10,11], it could represent a safer alternative to the first-generation DESs [12]. Also, the phosphorylcholine coating used for drug-elution from the ZES could be a safer non-inflammatory alternative to the polymers used for sirolimus-eluting and paclitaxel-eluting stents [13,14].

In the current study, based on data from the Western Denmark Heart Registry (WDHR), we used the sirolimus-eluting Cypher™ stent (SES) as the comparator stent. Intravascular ultrasound and angiography studies have shown that the SES reduced angiographic late lumen loss and neointima formation assessed by intravascular ultrasound to a level below detection with these modalities [1,15-17]. Preclinical studies showed that both ZES and SES were associated with delayed endothelialisation in a rabbit model [18], and that ZES had a higher grade of inflammation than SES at 30 days but less inflammation at 90 and 180 days in a porcine model [19]. Findings from randomised studies, the largest being the Danish Organisation for Randomized Trials with Clinical Outcome (SORT OUT) III trial, indicated that ZES, as compared to SES, had lower efficacy and was associated with increased risk of early stent thrombosis and all-cause mortality. As the external validity of randomised trials is limited by selection bias, it is important that data from randomised trials can be reproduced in observational studies [20]. The present study used the WDHR to compare ZES and SES in order to see if the randomised SORT OUT III data could be confirmed in a large cohort of consecutive patients.

## Methods

### Data extraction

Data for this population-based cohort study were obtained from the WDHR and national databases. The WDHR contains patient- and procedure-specific information on coronary interventions performed at the three coronary intervention centers in Western Denmark (Aarhus University Hospital, Skejby, Aarhus University Hospital, Aalborg, and Odense University Hospital). The national databases used in the study included the Danish Civil Registration System, the National Registry of Causes of Death, and the Danish National Registry of Patients, all of which cover the entire region’s population (approximately 3 million inhabitants, 55% of the Danish population). A detailed description of these databases has been published previously [21].

Between August 1, 2005 and October 1, 2007 10,992 patients were treated with percutaneous coronary intervention (PCI) in the region covered by the WDHR. The study cohort consisted of 6,122 (56%) patients with successful implantation of either the ZES (Endeavor, Medtronic, Santa Rosa, Ca) or the SES (Cypher Select or Cypher Select+; Cordis, Johnson & Johnson, Warren, NJ). For each patient we included only the first PCI procedure performed during the study period (the index procedure). Other drug-eluting stents or a combination of ZES/SES/other stents were utilized in 1,050 patients (10%), bare metal stents in 2,125 patients (19%), and balloon angioplasty or other interventions in 1,695 patients (15%). One-third of the patients included in the present study (n = 1,868; 31%) were randomised to ZES or SES as part of the SORT OUT III study, which enrolled patients between January 2006 and August 2007.

Post-PCI antiplatelet regimens included lifelong acetylsalicylic acid (75 mg daily) and clopidogrel with a loading dose of 300 mg or 600 mg followed by 75 mg daily. The recommended duration of clopidogrel treatment was 12 months during the entire study period.

The effectiveness parameter was clinically driven TLR, defined as any PCI including the stent or within 5 mm from the proximal or distal stent edge). Safety parameters included all-cause mortality, cardiac mortality, late myocardial infarction (MI; >30 days), and definite stent thrombosis.

We used the Academic Research Consortium definition of definite stent thrombosis [22]. We defined new MIs as hospitalization for MI occurring >30 days after the index PCI [23] based on admissions and readmissions for MI (ICD-10 codes I21-I21.9) identified from the Danish National Registry of Patients [21]. We used the original death certificates obtained from the National Registry of Cause of Deaths to classify deaths according to their underlying cause [21]. We ascertained all repeat coronary interventions (including balloon angioplasty, stent implantation, and coronary artery bypass grafting) from the WDHR. These re-interventions were divided into target vessel revascularization (TVR) and non-TVR. An endpoint committee examined all TVRs and identified all TLRs. Subsequently, all TLRs caused by stent thrombosis were identified by review of the angiogram and patient files, and the clinical presentation of these stent thromboses was identified and classified as STEMI, nonSTEMI, unstable angina pectoris, or stable angina pectoris.

We retrieved data from the WDHR on potential predictors of subsequent cardiovascular events, including patient, procedure, and lesion characteristics. We also obtained the complete hospitalization history since 1977 for each patient from the Danish National Registry of Patients until the date of stent implantation, and computed the patients’ Charlson Comorbidity Index scores, based on 19 major disease categories [24].

### Statistical analysis

We computed Aalen-Nelson curves for patients and lesions and used the life-table method to compute the cumulative incidence of each outcome. We used Cox proportional hazard regression to compute HRs as a measure of the relative risks for each outcome. Since the hazards were not proportional throughout the follow-up period, we computed HR estimates within separate time windows, for which the proportionality assumption held. The HRs in these sub-analyses reflected the risk among patients alive and at risk of a given outcome at the start of each time period. In regression analyses at the patient level, we controlled for age, sex, Charlson Comorbidity Index score (3 comorbidity levels), diabetes mellitus, PCI indication (STEMI, nonSTEMI/unstable angina pectoris, stable angina pectoris, or other), procedure time, number of lesions treated, total stent length (*i.e*., the combined length of stents in all treated lesions), and total number of stents implanted. In the lesion-specific analyses (stent thrombosis and TLR) we adjusted for age, sex, diabetes mellitus, PCI indication, procedure time, and stent length (*i.e*., the combined length of stents used to treat a specific lesion). The subgroup analyses of patients with and without diabetes were controlled for the same parameters except diabetes.

Depending on whether data conformed to a normal distribution, continuous variables were compared by use of the two-sample *t*-test or the Mann–Whitney test. Categorical variables were compared using the chi-square test. We used SAS software version 9.2 (SAS Institute Inc., Cary, NC, USA) to analyse the data.

## Results

Among patients included in this study, 2,282 patients with 3,090 lesions were treated with ZES and 3,840 patients with 5,095 lesions received SES. All patients were followed to November 10, 2007, *i.e.,.* for at least 40 days and up to a maximum of 823 days). The average follow-up time was 411 days (ZES: 342 days; SES: 452 days). Data on cause of death were not available for 59 patients.

Baseline patient and procedure characteristics presented in Table 1, and lesion characteristics presented in Table 2, showed differences between the ZES and SES groups. The most important were older age, longer procedure times, higher comorbidity scores, and differences regarding vessel and lesion types in the ZES group.

**Table 1:** Patient and procedure characteristics of patients treated with zotarolimus-eluting (ZES) or sirolimus-eluting stents (SES)

	***ZES N = 2,282***	***SES N = 3,840***	***P***
Age*	67 (59-75) years	65 (57-73) years	<0.0001
Male gender	1,645 (72%)	2,869 (75%)	0.024
Family history	807 (44%)	1,455 (46%)	0.17
Smoking	592 (34%)	1,121 (37%)	0.039
Diabetes mellitus	342 (15%)	602 (16%)	0.41
Hypertension	999 (54%)	1,573 (49%)	0.001
Previous coronary artery bypass operation	173 (9%)	279 (9%)	0.53
Previous percutaneous coronary intervention	735 (34%)	1,184 (32%)	0.25
Previous myocardial infarction	793 (37%)	1,268 (35%)	0.20
Lipid lowering treatment	1,217 (66%)	1,995 (63%)	0.027
Procedure time*	23 (15-36) min	21 (14-34) min	0.0007
Use of GPIIb/IIIa inhibitors	626 (27%)	1143 (30%)	0.051
Number of treated lesions*	1 (1-2)	1 (1-2)	0.175
Number of stents >1	514 (22.5%)	771 (20.1%)	0.023
Total stent length*	18.0 (14.0 - 24.0) mm	18.0 (13.0 - 26.0) mm	0.070
Indication for percutaneous coronary intervention			0.025
Stable angina pectoris	924 (41%)	1,610 (42%)	
Unstable angina pectoris/non ST-segment elevation myocardial infarction	763 (33%)	1,158 (30%)	
ST-segment elevation myocardial infarction	511 (22%)	946 (25%)	
Other	84 (4%)	126 (3%)	
Charlson’s comorbidity index score			0.0003
0	1,368 (60%)	2,475 (65%)	
1-2	725 (32%)	1,125 (29%)	
3+	189 (8%)	230 (6%)	

* Median (25%-75% quartiles).

**Table 2:** Lesion characteristics of patients treated with zotarolimus-eluting (ZES) or sirolimus-eluting (SES) stents

	**ZES N = 3,090 lesions**	**SES N = 5,095 lesions**	***P *Value**
Vessel			<0.0001
LM	99 (3.2%)	158 (3.1%)	
LAD	1,255 (41%)	2,388 (47%)	
LCX	743 (24%)	1,154 (23%)	
RCA	991 (32%)	1394 (27%)	
Sapheneous vein graft	36 (0.9%)	44 (1.2%)	0.18
Restenotic lesion	87 (2.8%)	74 (1.5%)	<0.0001
Stent thrombosis lesion	28 (0.9%)	24 (0.5%)	0.0076
Lesion length*	12 (10-20) mm	15 (10-20) mm	0.027
Lesion type			0.0008
A	659 (21%)	1,100 (22%)	
B1	823 (27%)	1,542 (30%)	
B2	629 (20%)	1,014 (20%)	
C	979 (32%)	1,439 (28%)	
Stent length^†^	19.9 ± 11.9 mm	20.2 ± 11.9 mm	<0.0001
Number of stents >1	581 (18.8%)	837 (16.4%)	0.006

* median (25%-75%). ^†^ mean ± standard deviation.

**Figure 1:**
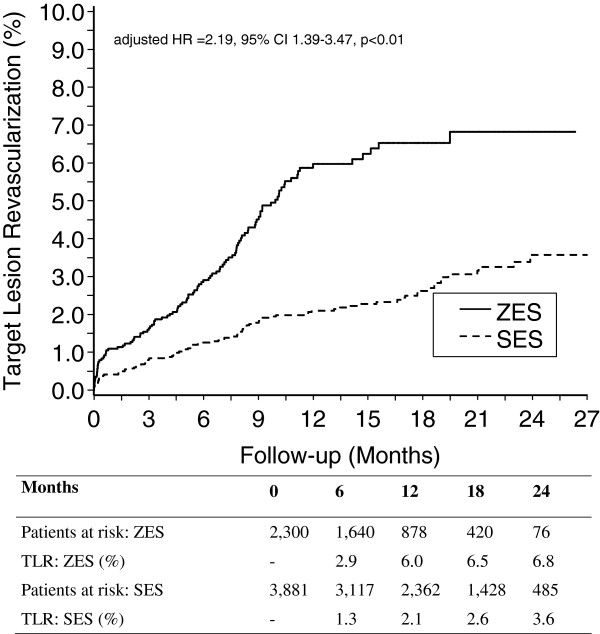
**Target lesion revascularization after ZES and SES implantation in Western Denmark.** Clinically driven target lesion revascularization among patients treated with ZES or SES.

**Figure 2:**
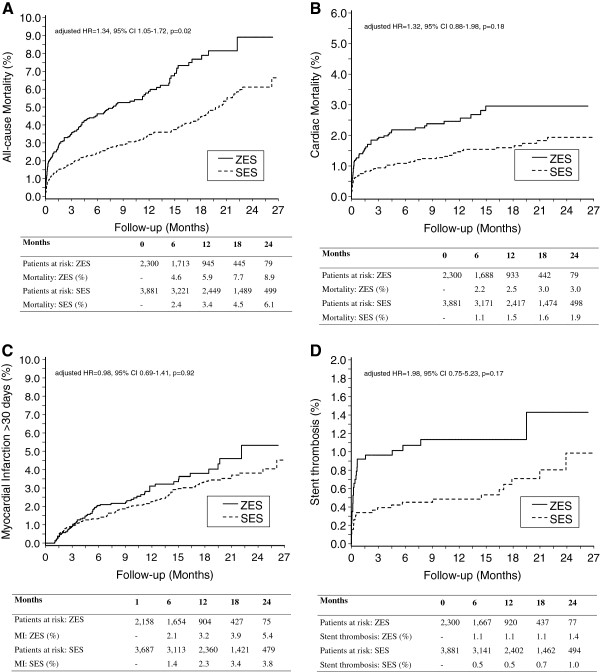
**Safety Outcomes of ZES and SES in the Western Denmark Heart Registry.** Risk of all-cause mortality (**A**), cardiac mortality (**B**), late (>30 days) myocardial infarction (**C**), and stent thrombosis (**D**) among patients treated with ZES or SES.

The outcome data are provided in Table 3 and the main outcomes are depicted in Figures 1 and 2. TLR (5.3% vs. 1.9%; adjusted hazard ratio [HR] 2.16, 95% confidence intervals [CI] 1.36-3.42; *p* = 0.001) was more than twice as common in the ZES group at the patient level, a finding confirmed at the lesion level of analysis. The safety parameters of all-cause mortality (6.3% vs. 3.3%; adjusted HR 1.35, 95% CI 1.05-173; *p* = 0.018), stent thrombosis at the lesion level (1.0% vs. 0.4%; adjusted HR 2.21, 95% CI 1.26-3.87; *p* = 0.006), but not stent thrombosis at the patient level (1.2% vs. 0.5%; adjusted HR 2.01, 95% CI 0.76-5.34; *p* = 0.159) were increased in the ZES group as compared to the SES group. This was apparently due to an excess of both early (day 0 to 30) mortality and early stent thrombosis. There were no significant differences between the two groups in late safety parameters (day 31 to 823).

**Table 3:** Hazard ratio (HR) estimates of adverse events among patients treated with zotarolimus-eluting stents (ZES) or sirolimus-eluting stents (SES) in western denmark, August 2005 to October 2007

**Endpoint**	**Period (days)**	**Patients with ZES**		**Patients with SES**		Adjusted HR* (95% CI)	***P *Value**
		***No. of events***	***No./100 person-year***	***No. of events***	***No./100 person-year***		
*Patient level analysis*		*N = 2,282*		*N = 3,840*			
Death	0-823	135	6.3	159	3.3	1.35 (1.05-1.73)	0.018
	0-30	57	31.0	50	16.0	1.66 (1.10-2.50)	0.016
	31-365	62	4.1	70	2.4	1.19 (0.82-1.73)	0.388
	366-823	16	3.5	39	2.5	1.26 (0.67-2.34)	0.475
Cardiac death	0-823	57	2.7	59	1.2	1.33 (0.89-2.00)	0.183
	0-30	32	17.4	29	9.3	1.39 (0.80-2.41)	0.229
	31-365	21	1.4	23	0.8	1.17 (0.59-2.32)	0.711
	366-823	4	0.9	7	0.4	1.36 (0.31-5.95)	0.687
Noncardiac death	0-823	53	2.5	66	1.4	1.27 (0.87-1.87)	0.217
	0-30	16	8.7	20	6.4	1.32 (0.65-2.66)	0.467
	31-365	31	2.1	34	2.1	1.25 (0.75-2.09)	0.397
	366-823	6	1.3	12	0.8	1.39 (0.50-3.89)	0.532
Myocardial infarction	31-823	66	3.5	108	2.5	0.98 (0.69-1.42)	0.964
	366-823	9	2.1	30	2.0	0.99 (0.43-2.28)	0.992
Target lesion revascularization	0-823	110	5.3	91	1.9	2.16 (1.36-3.42)	0.001
Stent thrombosis	0-823	26	1.2	24	0.5	2.01 (0.76-5.34)	0.159
	0-30	21	11.5	13	4.2	3.62 (0.92-14.2)	0.065
	31-365	4	0.3	5	0.2	1.17 (0.18-7.39)	0.870
	366-823	1	0.2	6	0.4	Not calculable	-
*Lesion level analysis*		*N = 3,090*		*N = 5,095*			
Target lesion revascularization	0-823	115	4.0	96	1.5	2.48 (1.87-3.27)	<0.0001
Stent thrombosis	0-823	28	1.0	24	0.4	2.21 (1.26-3.87)	0.006
	0-30	23	9.3	13	3.1	2.75 (1.38-5.47)	0.004
	31-365	4	0.2	5	0.1	1.47 (0.39-5.57)	0.568
	366-823	1	0.2	6	0.3	1.22 (0.14-10.9)	0.857

*The *patient level analyses* were adjusted for age, sex, Charlson Comorbidity score, diabetes mellitus, PCI indication, procedure time, no. of treated lesions, total stent length, and total no. of stents. The *lesion level analyses* were adjusted for age, sex, diabetes mellitus, PCI indication, procedure time, and stent length.

^†^ Defined as late (>30 days) definite stent thrombosis, myocardial infarction, or death from any cause.

Outcomes stratified by presence/absence of diabetes are presented in Table 4. Patients without diabetes treated with a ZES had increased risk of target lesion revascularization (TLR; 4.6% vs. 2.0%; HR 2.14, 95% CI 1.29-3.54; *p* = 0.0032) and early definite stent thrombosis at the lesion level (3.4% vs. 1.4%; HR 1.97, 95% CI 1.11-3.48, p = 0.0202). All other assessed parameters did not differ significantly. Patients with diabetes treated with a ZES had increased risk of death (9.9% vs. 4.6%, adjusted HR 2.03, 95% CI 1.21-3.42; *p* = 0.0076), cardiac death (5.4% vs. 1.5%; adjusted HR 3.54, 95% CI 1.59-7.90; *p* = 0.0020), and TLR (9.8% vs. 1.8%; adjusted HR 3.64, 95% CI 1.10-12.1; *p* = 0.0350). Lesion level analyses confirmed the findings for TLR and indicated a higher risk of stent thrombosis in the ZES group.

**Table 4:** Hazard ratio (hr) estimates of adverse events among patients with and without diabetes treated with cypher or endeavour stents

**Endpoint**	**Period (days)**	**Diabetic patients**		Adjusted HR* (95% CI)	**P value**	**Non diabetic patients**		Adjusted HR* (95% CI)	**P value**
**(no(no/person year))**		**Endeavour**	**Cypher**			**Endeavour**	**Cypher**		
Patient level analysis*		N = 342	N = 602			N = 1936	N = 3207		
Death	0-823	31 (9.9)	36 (4.6)	2.03 (1.21-3.42)	0.0076	102 (5.6)	122 (3.2)	1.20 (0.90-1.60)	0.20
	0-30	12 (43.9)	10 (20.5)	2.13 (0.88-5.15)	0.0942	43 (27.5)	40 (15.3)	1.52 (0.96-2.42)	0.077
	31-365	15 (6.9)	18 (3.9)	1.69 (0.80-3.58)	0.1701	47 (3.7)	51 (2.1)	1.09 (0.71-1.68)	0.70
Cardiac death	0-823	17 (5.4)	12 (1.5)	3.54 (1.59-7.90)	0.0020	38 (2.1)	46 (1.2)	0.97 (0.60-1.58)	0.91
	0-30	8 (29.3)	4 (8.2)	3.71 (1.03-13.4)	0.0449	22 (14.1)	25 (29.6)	1.09 (0.58-2.03)	0.79
	31-365	8 (3.7)	7 (1.5)	3.38 (1.10-10.4)	0.0341	13 (1.0)	15 (0.6)	0.70 (0.29-1.72)	0.44
Noncardiac death	0-823	12 (3.8)	14 (1.8)	1.65 (0.72-3.79)	0.2347	41 (2.3)	52 (1,3)	1.19 (0.77-1.83)	0.44
	0-30	4 (14.6)	6 (12.3)	1.22 (0.32-4.61)	0.7678	12 (7.7)	14 (5.4)	1.30 (0.56-3.02)	0.55
	31-365	7 (3.2)	7 (1.5)	1.67 (0.51-5.44)	0.3984	24 (1.9)	27 (1.1)	1.18 (0.66-2.10)	0.57
Myocardial infarction	30-823	54 (19.6)	86 (12.7)	1.24 (0.86-1.79)	0.2522	319 (20.4)	543 (16.4)	0.98 (0.85-1.13)	0.79
	0-30	41 (168)	68 (155)	1.24 (0.82-1.86)	0.3032	279 (206)	473 (210)	0.99 (0.85-1.16)	0.92
	31-365	10 (5.2)	14 (3.5)	0.89 (0.33-2.40)	0.8195	35 (3.2)	49 (2.4)	0.93 (0.56-1.55)	0.79
Composite safety end point^†^	30-823	31 (11.1)	48 (6.8)	1.45 (0.87-2.40)	0.1507	105 (6.5)	168 (4.7)	1.00 (0.76-1.31)	0.98
Target lesion revascularization	0-823	29 (9.8)	14 (1.8)	3.64 (1.10-12.1)	0.0350	81 (4.6)	76 (2.0)	2.14 (1.29-3.54)	0.003
In-stent restenosis	0-823	24 (8.0)	11 (1.4)	7.18 (1.59-32.4)	0.0103	60 (3.4)	56 (1.4)	1.97 (1.11-3.48)	0.020
Stent thrombosis	0-823	5 (1.6)	4 (0.5)	-	-	21 (1.2)	20 (0.5)	2.92 (0.95-8.93)	0.060
	0-30	4 (14.7)	1 (2.0)	-	-	17 (10.9)	12 (4.6)	5.61 (1.14-27.6)	0.034
	31-365	1 (0.5)	1 (0.2)	-	-	3 (0.2)	4 (0.2)	1.74 (0.23-13.2)	0.59
	366-823	0 (0.0)	2 (0.8)	-	-	1 (0.3)	4 0.3)	-	-
Lesion level analysis									
Target lesion revascularization	0-823	29 (7.3)	15 (1.4)	5.06 (2.65-9.69)	<.0001	86 (3.5)	80 (1.5)	2.08 (1.53-2.85)	<0.0001
In-stent restenosis	0-823	24 (6.0)	12 (1.1)	5.06 (2.48-10.3)	<.0001	63 (2.5)	60 (1.2)	2.08 (1.45-2.99)	<0.0001
Stent thrombosis	0-823	5 (1.2)	4 (0.4)	4.33 (1.02-18.4)	0.047	23 (0.9)	20 (0.4)	2.03 (1.10-3.76)	0.0234
	0-30	4 (11.2)	1 (1.5)	11.6 (1.10-122)	0.0413	19 (9.0)	12 (3.5)	2.40 (1.15-4.99)	0.0194
	31-365	1 (0.4)	1 (0.2)	1.84 (0.11-30.5)	0.6693	3 (0.2)	4 (0.1)	1.34 (0.29-6.08)	0.7074
	366-823	0 (0.0)	2 (0.5)	-	-	1 (0.2)	4 (0.2)	1.68 (0.17-16.4)	0.66

* The patient level analyses were adjusted for age, sex, Charlson Comorbidity score, PCI indication, procedure time, no. of treated lesions, total stent length, and total no. of stents. The lesion level analyses were adjusted for age, sex, PCI indication, procedure time, and stent length.

† Defined as late (>30 days) definite stent thrombosis, myocardial infarction, or death from any cause.

Stent thrombosis accounted for 50 of the 202 TLRs. These patients were admitted due to ST-segment elevation myocardial infarction [STEMI; n = 39 (78.0%)], nonSTEMI [n = 6 (12.0%)], and unstable angina pectoris [n = 5 (10.0%)].

## Discussion

The SORT OUT III trial reported that the ZES was inferior to SES regarding both efficacy and safety [25]. Although we attempted to randomise routine clinical care patients (“all-comers”) in SORT OUT III, we reported that the separate group of patients that were eligible, but for different reasons not randomised, had higher 30-day mortality than patients who entered the trial (3% vs. <1%). It is therefore of clinical importance to confirm the generalizability of the SORT OUT III trial by examination of the entire cohort of patients treated with a ZES or SES in Western Denmark. Our data confirm and extend the SORT OUT III results to all patients treated with ZES and SES in Western Denmark. As in the SORT OUT III trial, we found that compared with the SES, use of the ZES increased all-cause mortality, and produced twice as high rates of TLR and definite stent thrombosis at medium-term follow-up.

The finding that ZES has higher TLR rates than SES is concurrent with efficacy analyses based on intravascular ultrasound [11], optical coherence tomography [26], angiography [27], and with clinical results reported from a Swedish registry [28], a large Korean randomised comparison [29], a Korean randomised study in patients with ST-segment elevation [30], a German randomised study [31], and the SORT OUT III trial [25]. Although carefully performed analyses have shown that late lumen loss detected by angiography predicted ISR and TLR [32-34], it has been suggested that minor late loss differences in the lower end of the late loss scale may not lead to TLR differences [12,34]. Based on the current data, the reported in-stent late loss difference between the two stents seems large enough to produce a significant difference in clinically driven TLR.

Definite stent thrombosis constituted 25% of all TLRs, caused MI in 90% of patients experiencing this adverse event, and was the cause of all but one TLR-related STEMI. Even though TLR was a relatively rare event in the present cohort, definite stent thrombosis was a relatively frequent and high-risk cause of TLR. ZES was associated with higher rates of definite stent thrombosis and death during the first 30 days after implantation whereas we found no overall differences between the two study stents in late safety outcomes from day 31 on. In comparison to SES and other available drug-eluting stents, it is possible that the rapid elution of zotarolimus from the phosphorylcholine coating creates a milieu in the stented arterial wall that facilitates early stent thrombosis and thus explains the reported increased risk of early stent thrombosis [25,29]. This hypothesis is supported by preclinical data describing more vascular inflammation with the ZES than the SES at 30 days [19]. With regard to very late (>1 year) stent thrombosis, the phosphorylcholine coating and the larger neointima formation by the ZES may still reduce the risk of this adverse event when dual antiplatelet therapy is limited to aspirin only, but our current comparison to SES is inconclusive on this issue. A recent study by Serruys *et al*[35]. showed that a modified slow-release zotarolimus-eluting stent (the Resolute™ stent) was non-inferior to an everolimus-eluting stent. It may thus be release kinetics rather than type of drug that explains the clinical differences between ZES and SES implantation.

Patients with diabetes have a higher risk of adverse events after PCI, which includes an increased rate of TLR due to excessive neointima formation in diabetic patients [36]. Our results confirm this hypothesis. In comparison to SES, we found that ZES-treated patients without diabetes had a 2.1-fold higher risk of TLR while ZES-treated diabetic patients had a 3.6-fold higher risk. These results accord with results from a Swedish registry and a small randomized trial [28,37]. Efficacy differences between various DESs may thus be amplified in diabetic patients.

Our risk estimates are based on population-based data, largely ruling out referral and diagnostic biases. Adjustment for a broad range of important prognostic cardiovascular factors and the fact that 31% of patients were randomised to the study stent are likely to have reduced the risk of any major uncontrolled or residual confounding. Although our study is still limited by potential confounding by indication due to lack of randomization of the majority of patients, the strength of this study is that the results are consistent with the randomised SORT OUT III. The clinical outcome data were ascertained by use of registry data. In this regard it is important that the study was performed in a small European country with a stable population and an accurate registration of the vital status and cause of death of its citizens. All contacts with the Danish healthcare system are registered, allowing 100% follow-up. Underreporting of events (such as MIs or TLRs during visits abroad) and misclassifications are possible, but unlikely to have a major impact on our results, and equally unlikely to favour one of the two study stents. Another consideration is that the study’s follow-up time was too short to fully quantify possible risks. It is possible that longer follow-up would yield different long-term safety profiles for the two drug-eluting stents. Moreover, our results with the fast-release Endeavor™ ZES cannot be extrapolated to the newer slow-release Resolute™ ZES [35]. Finally, we do not have data on the duration of dual anti-platelet treatment for the two groups, which theoretically might have affected the results.

## Conclusions

We conclude that this observational study found that ZES is inferior to SES, thereby extending randomised results to a large cohort of consecutive patients.

## Abbreviation

CI: Confidence interval; DES: Drug-eluting stent; HR: Hazard ratio; MI: Myocardial infarction; PCI: Percutaneous coronary intervention; SES: Sirolimus-eluting stent; SORT OUT: Dani**S**h **O**rganisation for **R**andomised **T**rials with Clinical **OUT**come; STEMI: ST-segment elevation MI; TLR: Target lesion revascularization; TVR: Target vessel revascularization; WDHR: Western Denmark Heart Registry; ZES: Zotarolimus-eluting stent.

## Competing interests

Medtronic and Cordis have both supported research at the three departments of cardiology in Aalborg, Skejby, and Odense through unrestricted research grants.

## Authors’ contributions

MiM was responsible for the conception and design of the study. All authors except MoM and HTS contributed to the review of clinical events. MoM and HTS was responsible for the statistical analysis. MiM drafted the manuscript. All authors read and approved the final manuscript.

## Pre-publication history

The pre-publication history for this paper can be accessed here:

http://www.biomedcentral.com/1471-2261/12/84/prepub
